# Unmasking hidden pathways: how household air pollution and lifestyle are associated with quality of life in Yunnan outpatients

**DOI:** 10.3389/fpubh.2026.1796796

**Published:** 2026-05-27

**Authors:** Jianhui He, Bo Chen, Liping Wang, Zhen Yu, Jing Li

**Affiliations:** 1 Yunnan Provincial Key Laboratory of Public Health and Biosafety, School of Public Health, Kunming Medical University, Kunming, China; 2 Department of Foreign Languages, Kunming Medical University, Kunming, China

**Keywords:** comorbidity, health-related quality of life (HRQoL), household air pollutant exposures, non-communicable chronic diseases (NCDs), structural equation modeling (SEM)

## Abstract

**Background:**

Non-communicable diseases (NCDs) are a major health challenge in China. This study examined comorbidity prevalence among outpatients in Yunnan and its association with health-related quality of life (HRQoL).

**Methods:**

Using stratified cluster sampling, 5,978 outpatients (2019–2022) were recruited. Data on comorbidities, HRQoL (EQ-5D-5L), and sociodemographics were analyzed via structural equation modeling.

**Results:**

Mean age was 38.1 years; 56.8% female. Nearly half (49.1%) had ≥1 NCD. Mean HRQoL score was 0.89 ± 0.20. HRQoL was positively associated with socioeconomic status (*β* = 0.170, 95%CI: 0.140–0.199), Han ethnicity (0.052, 0.027–0.077), and preference for heavily seasoned foods (0.028, 0.006–0.051); negatively with age (−0.171, −0.190 to −0.143), addictive behaviors (−0.070, −0.094 to −0.046), divorce/widowhood (−0.082, −0.131 to −0.034), and NCDs (−0.040, −0.060 to −0.020). NCDs were positively associated with household fuel exposure (0.647, 0.615–0.679), age (0.143, 0.123–0.162), and addictive behaviors (0.075, 0.050–0.100); negatively with socioeconomic status (−0.064, −0.097 to −0.030) and Han ethnicity (−0.037, −0.064 to −0.010). Age, ethnicity, fuel exposure, addictive behaviors, and socioeconomic status had indirect associations with HRQoL via NCDs.

**Conclusion:**

Socioeconomic status, household fuel exposure, addictive behaviors, and demographics are significantly linked to NCDs and HRQoL. Interventions targeting modifiable factors are needed.

## 1 Introduction

The rising prevalence of non-communicable chronic diseases (NCDs) and their associated comorbidities has become a critical public health issue globally. NCDs, including cardiovascular diseases, cancers, chronic respiratory diseases, and diabetes, are responsible for 41 million deaths annually, accounting for 74% of all deaths worldwide (1, 2). Low- and middle-income countries (LMICs) bear a disproportionate burden, with 77% of global NCD deaths occurring in these settings (3). The economic impact is equally staggering, with NCDs projected to cost the global economy $47 trillion between 2010 and 2030 (4), while productivity losses in Europe alone exceed $514.5 billion annually (5). In China, the emergence of NCDs as the leading cause of death has been particularly rapid, with cardiovascular diseases, cancer, and chronic respiratory diseases accounting for more than 80% of all deaths by the early 2000s (6). By 2017, ischemic heart disease and stroke had become the top two causes of years of life lost in China, reflecting a profound epidemiological transition over three decades (7). Comorbidity, defined as the coexistence of two or more chronic conditions in an individual (8), compounds this burden by increasing healthcare utilization, reducing health-related quality of life (HRQoL), and elevating mortality rates. In China, the burden of NCDs is particularly pronounced in regions such as Yunnan Province, where socioeconomic disparities, household air pollutant exposures, and lifestyle factors are associated with the complexity of comorbidity patterns. For instance, a recent longitudinal study in rural Yunnan found that the prevalence of chronic obstructive pulmonary disease (COPD) was significantly higher among populations with lower socioeconomic status, underscoring the widening health inequality over the past decade (9). A systematic analysis of the Global Burden of Disease Study 2017 revealed that Yunnan exhibited higher age-standardized mortality rates from chronic respiratory diseases compared to more developed coastal provinces, underscoring the need for region-specific interventions (7). Understanding the interplay between comorbidities, HRQoL, and their determinants is essential for developing targeted interventions to improve health outcomes in outpatient populations.

Health-related quality of life (HRQoL) is a multidimensional construct that reflects an individual’s physical, mental, and social well-being. Previous studies have demonstrated that comorbidities are significantly associated with HRQoL, with the number and severity of chronic conditions being inversely related to quality of life (10, 11). However, the relationship between comorbidities and HRQoL is influenced by a range of factors, including socioeconomic status, lifestyle behaviors, and household air pollutant (HAP) exposures (11–14).

Age emerged as a significant factor associated with both lower health-related quality of life (HRQoL) and a higher burden of non-communicable diseases (NCDs) (15–17). Notably, its influence on HRQoL operates through dual pathways: a direct effect attributable to physiological decline, and a small but significant indirect effect mediated by increased NCD risk (16). This compounding effect of aging and multimorbidity underscores a growing challenge for health systems worldwide (17).

Sociodemographic factors are well-established determinants of health outcomes. Marital disruption, specifically divorce or widowhood, has been consistently associated with lower health-related quality of life (HRQoL) across various populations, including among individuals with chronic conditions and older adults (18, 19). This association is often attributed to the loss of social support and economic strain that frequently accompany such transitions, and may be partially explained by consequent changes in socioeconomic status (18, 19). Ethnic background has also been identified as a significant correlate of health. For instance, ethnic majority status (Han Chinese, in this context) has been linked to modestly better HRQoL and a lower burden of non-communicable diseases (NCDs) (20–23). These patterns underscore the importance of considering both marital status and ethnicity as fundamental social determinants that shape health disparities (20, 23).

Lower socioeconomic status (SES) has been consistently associated with poorer health-related quality of life (HRQoL) and higher comorbidity rates, largely attributable to limited healthcare access and adverse living conditions (24, 25). Research consistently demonstrates that SES is a key determinant of HRQoL, exhibiting the strongest direct positive association with it (25). This finding aligns with the broader global literature, which identifies SES as a fundamental driver of health disparities (26). Higher SES facilitates improved access to healthcare, healthier environments, nutritious food, and greater health literacy—all of which contribute to enhanced HRQoL (27, 28). Importantly, higher SES is also associated with a lower burden of non-communicable diseases (NCDs) (28).

Accumulating empirical evidence further underscores the significant role of HAP in chronic disease development. A recent ecological analysis from Iran demonstrated significant positive correlations between long-term HAP exposure and elevated prevalence of hypertension, diabetes mellitus, obesity, and high LDL cholesterol among older adults (29). Complementing these findings, a prospective cohort study in China revealed that solid fuel use for cooking was associated with a 70% higher risk of chronic kidney disease (OR 1.70; 95% CI: 1.07–2.70), with the strongest effects observed among women and individuals with lower education (30). The global burden of HAP-attributable disease remains substantial; a 2021 Global Burden of Disease study estimated that HAP exposure accounted for 111 million disability-adjusted life-years (DALYs) worldwide, representing 3.9% of all DALYs, with type 2 diabetes among the contributing conditions (31). Furthermore, a prospective analysis of UK Biobank data found that residential environments with abundant green-blue spaces were associated with lower risks of 41 chronic diseases across 12 categories, an effect partially mediated by reduced air pollutant concentrations (32). A 2026 study from Dalian, China, quantified the relative risks and disease burden of indoor pollutants, showing that CO exhibited the strongest association with ischemic heart disease risk (RR 1.13 at 4 mg/m^3^), while SO₂ and NO₂ exerted pronounced effects on acute asthma exacerbations (33). These studies collectively reinforce that HAP exposure constitutes a critical risk factor across multiple chronic disease pathways, supporting its inclusion in comprehensive models of HRQOL determinants.

Addictive behaviors, particularly tobacco smoking and hazardous alcohol consumption, are well-established determinants of both chronic disease development and health-related quality of life (HRQoL). A substantial body of evidence demonstrates that smoking is consistently associated with poorer health outcomes, including increased risk of cardiometabolic disorders, respiratory diseases, and various cancers (34–36). Systematic reviews have shown that smoking contributes significantly to socioeconomic inequalities in health, with an estimated median contribution of 19% to the social gradient in cardiometabolic disease and mortality (34). Furthermore, non-smokers consistently experience more healthy life years and better HRQoL throughout their lives compared to smokers (37). Alcohol consumption exhibits a more complex relationship with health outcomes. Hazardous or harmful alcohol use has been linked to increased symptom burden, including breathlessness and chronic cough, as well as lower physical and mental HRQoL scores (34). Among individuals with chronic conditions, such as rheumatic and musculoskeletal diseases, moderate to high alcohol consumption is associated with increased disease activity and poorer prognostic outcomes (36). Evidence from Chinese older populations similarly identifies excessive drinking as a significant negative predictor of HRQoL (35). The cumulative impact of these behaviors is particularly pronounced when they co-occur. Multiple unhealthy behaviors contribute more substantially to health inequalities than individual behaviors alone (34). Both smoking and harmful alcohol use have been shown to exacerbate the effects of existing chronic conditions, leading to greater symptom severity and reduced quality of life (34, 38). These findings underscore the critical importance of addressing addictive behaviors in public health strategies aimed at reducing chronic disease burden and improving population HRQoL.

Dietary patterns characterized by high consumption of fats, salt, and preserved foods have been consistently associated with increased chronic disease risk and diminished health-related quality of life (HRQoL). High salt intake is a well-established risk factor for hypertension and cardiovascular disease, with each 1 g/day increase in sodium raising systolic blood pressure by 0.60 mmHg and increasing cardiovascular disease and stroke risks by 4 and 6%, respectively (39). Excessive salt consumption has been linked to increased risks of cardiovascular disease (RR = 1.13), hypertension (OR = 1.33), and stroke (OR = 1.34) (39). High dietary fat intake, particularly from unhealthy sources, contributes to obesity, metabolic disorders, and cardiovascular risk (40). While the relationship between total fat and chronic disease outcomes remains complex, dietary patterns high in processed and red meat, discretionary fats, and fried foods are consistently linked to adverse health outcomes (40). Preserved vegetable consumption, common in many Asian diets, has been specifically associated with gastrointestinal cancer risk. Evidence from large prospective cohorts indicates that salted vegetable consumption is positively associated with stomach cancer risk (HR = 1.17), while sour pickled vegetables show dose–response relationships with oesophageal cancer risk (HR = 1.35 for daily consumption) (41, 42). These associations likely reflect the combined effects of high sodium content and potential carcinogenic compounds formed during preservation. Beyond specific disease outcomes, unhealthy dietary patterns characterized by high intake of fats, salt, and processed foods have been inversely associated with both physical and mental components of HRQoL (43). The cumulative burden of diet-related chronic conditions, mediated through hypertension, metabolic dysfunction, and cancer risk, ultimately contributes to reduced quality of life and increased healthcare utilization.

Structural Equation Modeling (SEM) is a powerful statistical tool for examining complex relationships among multiple variables, including comorbidities, HRQoL, and their determinants (44). Unlike traditional regression models, SEM allows for the simultaneous analysis of direct and indirect pathways, providing a more comprehensive understanding of how various factors may be interrelated health outcomes. This study employs SEM to evaluate comorbidity patterns among outpatients in Yunnan Province, China, and to investigate their associations with HRQoL and other relevant factors. By focusing on a region with unique socioeconomic and environmental characteristics, this study addresses a critical gap in the literature and provides novel insights into the factors associated with comorbidity and HRQoL.

Age, socioeconomic status, and key sociodemographic factors are fundamental determinants of health-related quality of life (HRQoL) and non-communicable disease (NCD) burden. Age exerts dual effects through physiological decline and increased multimorbidity risk. Marital disruption and ethnic background shape health disparities through social support mechanisms and cultural determinants. Socioeconomic status demonstrates strong positive associations with HRQoL by enabling healthcare access, healthier environments, and health literacy. Household air pollution represents a critical modifiable environmental exposure linked to hypertension, diabetes, and respiratory conditions. Tobacco smoking and hazardous alcohol consumption consistently predict poorer health outcomes, reduced healthy life years, and increased symptom burden, particularly when co-occurring. Unhealthy dietary patterns—characterized by high salt, fat, and preserved food intake—contribute to hypertension, cardiovascular disease, and gastrointestinal cancers, subsequently diminishing both physical and mental HRQoL components. These interrelated determinants collectively inform comprehensive models of population health.

**Figure 1:**
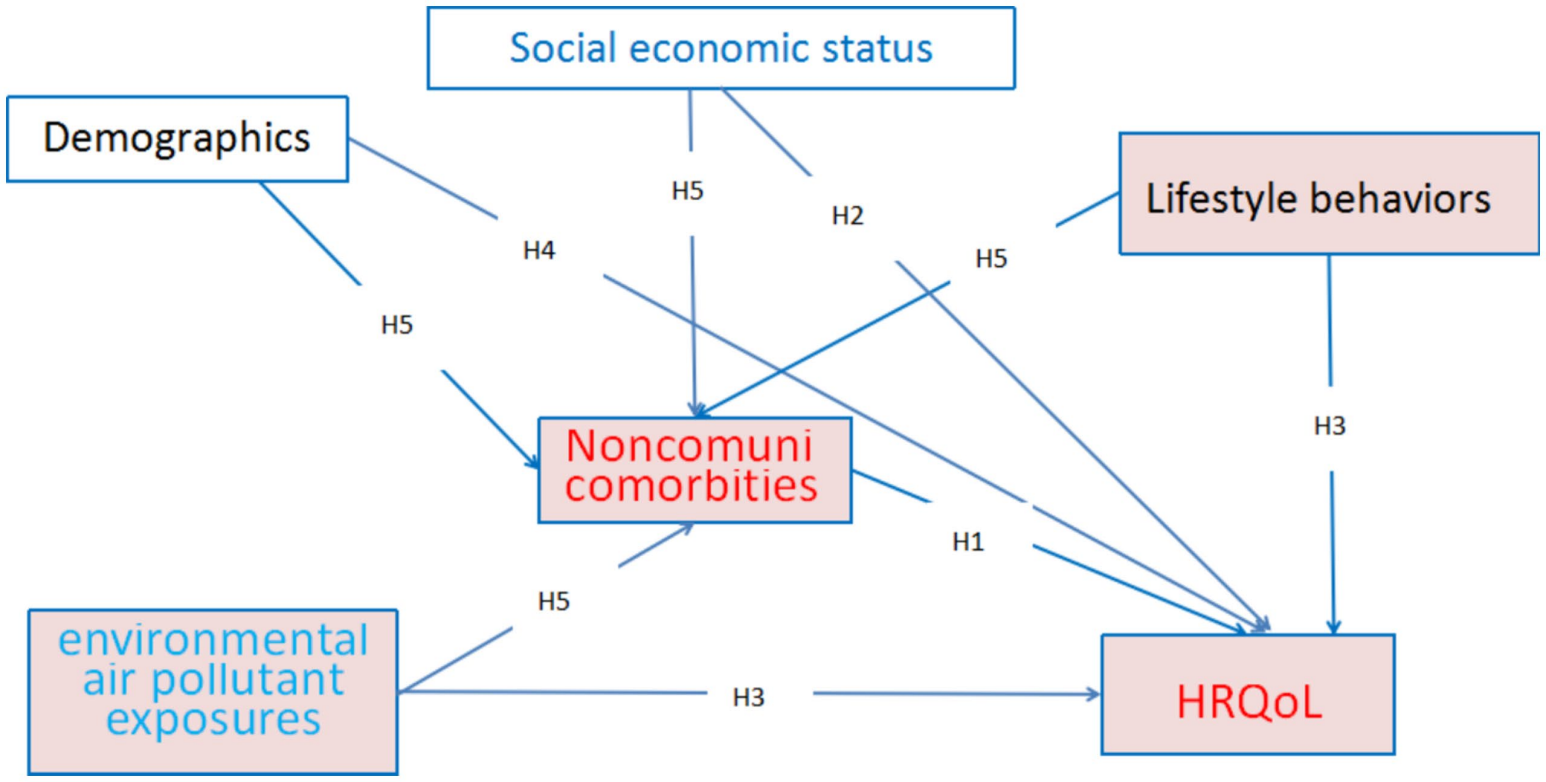
Hypotheses of the study (diagram showing pathways between socioeconomic, behavioral, environmental, and clinical factors influencing HRQoL and NCDs).

Building on existing literature and leveraging SEM, this study proposes novel hypotheses: (1) Comorbidity status (e.g., the presence of one or more NCDs) will negatively associated with HRQoL (EQ-5D-5L and self-rated health). (2) Socioeconomic status will associated with NCDs and associated with HRQoL. (3) Lifestyle behaviors (e.g., heavy taste, alcohol, smoking) and environmental air pollutant exposures (e.g., household fuel) will negatively associated with HRQoL and associated with NCD risk. (4) Demographic factors (e.g., age, ethnicity) will be associated with comorbidity status and HRQoL. (5) Comorbidity status is a important mediator in the associations between age, ethnicity, household fuel exposure, addictive behaviors, socioeconomic status and HRQoL (Figure 1).

By testing these hypotheses, this study aims to provide a comprehensive understanding of the complex interplay between comorbidity, HRQoL, and their determinants, offering evidence-based insights for targeted public health interventions in Yunnan Province and similar regions.

## 2 Materials and methods

### 2.1 Study setting

This cross-sectional study employed a questionnaire-based survey to assess quality of life among patients attending comprehensive medical institutions in Yunnan Province between June 2019 and March 2022. A multi-stage stratified sampling design was used to recruit outpatient participants. The sampling process proceeded as follows: First, stratification by regional economic status. All 16 prefectures in Yunnan were stratified into two strata based on their 2016 Gross Domestic Product (GDP): high-GDP and low-GDP. From each stratum, three prefectures were randomly selected. The high-GDP stratum included Kunming, Qujing, and Honghe; the low-GDP stratum included Dali, Lijiang, and Pu’er, resulting in a total of six prefectures. Second, stratification by healthcare institution function. Within each selected prefecture, all public healthcare institutions were stratified into two categories based on primary function: curative (hospitals) and preventive (e.g., centers for disease control and prevention, maternal and child health centers). A complete list of all formally employed healthcare professionals working in these institutions within the selected prefectures was compiled. Third, random selection of healthcare professionals (serving as the sampling frame for patients). The total sample size of healthcare professionals was set at 228. The number of professionals to be sampled from each stratum (defined by prefecture and institution type) was allocated proportionally to the total number of professionals in that stratum. Individual professionals were then randomly selected from a complete name list within each stratum using simple random sampling. To ensure representativeness and avoid clustering within departments, the selection process was further refined by: (1) stratifying by sex to achieve a balanced sample of 114 males and 114 females, and (2) applying a rule that only one professional was selected from any single department. This process was repeated until 228 unique professionals from 228 different departments across the six prefectures were recruited. Fourth, recruitment of outpatient participants. The final study participants were outpatients seeking care from the departments of the 228 selected healthcare professionals. All eligible outpatients who visited these specific departments during the study period were invited to participate. The sampling unit for analysis was the individual outpatient, nested within the departments and prefectures defined by the sampling strategy. The findings are generalizable to the outpatient population seeking care in public curative and preventive healthcare facilities in the six selected prefectures, which represent the diverse economic regions of Yunnan Province. A total of 5,978 valid questionnaires were collected from eligible participants.

### 2.2 Measurements

Self-designed questionnaires were utilized to collect data on sociodemographic characteristics, comorbidities, healthcare utilization, and exposure to environmental toxic substances, as well as legal substance use (alcohol consumption and tobacco smoking). Health-related quality of life (HRQoL) was assessed using the Chinese version of the EQ-5D scale.

### 2.3 Variables

Composite indices were constructed for the relevant factors as an alternative to factor analysis, aiming to reduce model complexity and enhance interpretability and clarity. All composite indices were constructed by first standardizing each raw indicator into a *Z*-score using the formula *Z* = (*X* − *μ*)/*σ* and then averaging the standardized scores of the constituent indicators. For a composite index comprising *k* indicators, the score is given by $\frac{1}{k}\sum_{i=1}^{k}Z_{i}$ . Following this procedure: Quality of Life (QOL) was operationalized as the average of two standardized indicators: self-assessed health ratings and utility values. Socioeconomic status (SES) was derived analogously as the mean of three standardized indicators: income, education, and occupational status (Based on a hierarchical ordering of social status, ordinal scores of 0, 1, 2, 3, and 4 were assigned to the unemployed, students, farmers, freelancers, and those in other fixed occupations, respectively). The addictive behavior index was constructed similarly by combining standardized measures of smoking (status and quantity) and drinking (status, frequency, and quantity) behaviors. The observed variables included: age, measured as a continuous variable; gender and ethnicity, both coded as binary variables (Minority = 1, Non-Minority = 0); and marital status, categorized as married versus unmarried, with the unmarried category encompassing cohabiting, divorced, or widowed individuals. The non-communicable disease comorbidity score was calculated by summing the number of chronic diseases, with each condition assigned a score of 1. Household air pollution exposure was primarily represented by cooking fuels, with data collected on the use of firewood, coal, natural gas, and electricity as indicators of exposure to air pollutants. One point was assigned for each type of fuel used, and the cumulative points were summed to obtain the household air pollutant exposure score. Similarly, the heavy-taste score was constructed by aggregating scores for the frequency of pickled food intake (assigned a value of 1 if consumed more than once per week, otherwise 0), the frequency of high-oil food intake (assigned a value of 1 if consumed more than once per week, otherwise 0), and salt intake compared to others (assigned a value of 0 if less salty than others, and a value of 1 if equally salty or saltier than others).

### 2.4 Data management and statistical analysis

Data management were used by Epidata 3.1, and data analysis were conducted by R-4.2.3. Chi-square tests and rank sum tests were used for comparison of risk levels in univariate analysis. Structural equation model (SEM) analysis was employed to explore the complex relationships among comorbidities, HRQoL, and their determinants. The model was constructed based on the theoretical framework and hypotheses outlined in the introduction (see Figure 1). The specific settings and construction process were as follows: First, the model was specified as a path model with latent and observed variables. Health-related quality of life (HRQoL) was treated as a latent endogenous variable, operationalized by two observed indicators: the EQ-5D-5L utility score and the self-rated health score. Socioeconomic status (SES) was also specified as a latent endogenous variable, indicated by standardized scores for income, education, and occupational status. The comorbidity score (a count of chronic conditions) was modeled as an observed endogenous variable, while also serving as a mediating variable. Exogenous variables included age (observed, continuous), ethnicity (observed, binary), marital status (observed, binary), household air pollution (HAP) exposure score (observed, continuous), addictive behavior index (observed, continuous), and heavy-taste score (observed, continuous). The construction of the composite scores followed the procedures detailed in Section 2.3. The HAP exposure score was calculated as the sum of four binary indicators (use of firewood, coal, natural gas, and electricity), yielding an integer score ranging from 0 to 4. The heavy-taste score was similarly obtained by summing three binary indicators (frequency of pickled food intake, frequency of high-oil food intake, and comparative salt intake), with a possible range of 0 to 3. The addictive behavior index was derived by averaging the standardized scores of smoking (status and quantity) and drinking (status, frequency, and quantity) behaviors, resulting in a continuous measure. Although the HAP and heavy-taste scores are technically discrete, their distributions were sufficiently dispersed (with variances > 0.5 and skewness < |1|) to be reasonably approximated as continuous variables in the SEM framework. This approximation facilitates model parsimony while preserving the essential information contained in these exposures. Second, model identification was ensured by applying the t-rule and scaling the latent variables by fixing the factor loading of one indicator per latent construct to 1. The model was over-identified, allowing for the estimation of fit statistics. Third, given the presence of categorical observed variables (e.g., binary ethnicity and marital status) and ordinal indicators for some latent constructs, parameters were estimated using the robust weighted least squares mean and variance adjusted (WLSMV) estimator. This estimator is specifically designed for models with categorical data and does not require the assumption of multivariate normality, thereby providing more reliable standard errors, test statistics, and fit indices. The WLSMV estimation was implemented by treating the composite scores (HAP exposure, addictive behavior, heavy taste) as continuous variables, while specifying ethnicity and marital status as categorical. The estimator utilizes a polychoric correlation matrix for ordinal indicators and a diagonal weight matrix, and it remains robust to the mild non-normality that may arise from the discrete nature of the composite scores. Fourth, to assess the model fit, we used a combination of absolute and incremental fit indices suitable for WLSMV estimation: the chi-square statistic divided by its degrees of freedom (*χ*^2^/df, with values < 3 indicating acceptable fit), the Root Mean Square Error of Approximation (RMSEA; acceptable fit < 0.06), the Comparative Fit Index (CFI; acceptable fit > 0.90), and the Adjusted Goodness-of-Fit Index (AGFI; acceptable fit > 0.80). Robust versions of these indices (e.g., robust CFI, robust RMSEA) were used as provided by the software. Fifth, to test the hypothesized mediation effects (e.g., the role of comorbidity status in mediating the relationships between age, SES, lifestyle factors, and HRQoL), we conducted a bootstrapping procedure with 5,000 resamples to generate bias-corrected 95% confidence intervals for the indirect effects. An indirect effect was considered statistically significant if the confidence interval did not include zero. All statistical tests were two-sided, and *p* < 0.05 was considered statistically significant. For variables with missing values ≤20%, mean or median imputation was applied based on the nature of the data. Variables with missing values exceeding 20% were excluded from the analysis.

## 3 Results

### 3.1 Distribution of socio-demographic characteristics and study variables

The study included 5,978 outpatients, comprising 2,580 males (43.2%) and 3,398 females (56.8%). The mean age was 38.1 years. Participants included 35.5% from ethnic minority groups and 2.9% who were divorced. Regarding socioeconomic status, 65.3% had attained a junior high school education or higher, 89.2% possessed medical insurance, and 57.1% reported a monthly family income exceeding 5,000 CNY. Only 6.5% reported no preference for heavily seasoned foods. Key continuous variables are summarized as follows (mean ± SD or median (IQR)): smoking index, −0.448 (−0.448, −0.448); drinking index, −0.493 (−0.493, −0.493); self-rated health status score, 76.8 ± 15.7; quality of life utility score, 0.892 ± 0.197; number of non-communicable disease comorbidities, 0 (0, 2); household fuel exposure level, 1 (1, 3) (Table 1).

**Table 1:** Descriptive statistics of study variables.

Variables	*n*	Mean ± SD or Median (P25, P75) or %
Demographic characteristics		
Age (years)	5,978	38.1 ± 19.0
Gender		
Female	3,398	56.8
Male	2,580	43.2
Ethnicity		
Minority	2,123	35.5
Han	3,855	64.5
Marital status		
Divorced/Widowed	171	2.9
Others	5,807	97.1
Education level		
Illiterate	687	11.5
Primary school	1,387	23.2
Junior/Senior high school	2,495	41.7
College and above	1,409	23.6
Family monthly income (RMB)		
0	205	3.4
<800	245	4.1
800–2000	754	12.6
2000–5,000	1,364	22.8
5,000–8,000	1,606	26.9
>8,000	1804	30.2
Health-related factors		
Health-related quality of life		
Self-rated health score	5,978	76.8 ± 15.7
EQ-5D utility score	5,978	0.892 ± 0.197
Number of NCDs	5,978	0 (0, 2)
Behavioral and environmental exposures		
Smoking index^a^	5,978	−0.448 (−0.448, −0.448)
Drinking index^b^	5,978	−0.493 (−0.493, −0.493)
Household fuel exposure score^c^	5,978	1 (1, 3)
Heavy taste score^d^		
0 (none)	386	6.5
1 (one type)	1,067	17.8
2 (two types)	1785	29.9
3 (three types)	2,740	45.8
Healthcare access		
Medical insurance covering disease payment		
Yes	5,333	89.2
No	645	10.8

Data are presented as mean ± SD, median (P25, P75), or frequency (%).

a Smoking index: composite score based on smoking status and daily smoking amount.

b Drinking index: composite score based on drinking status, frequency, and amount.

c Household fuel exposure score: sum of fuel types used (firewood, coal, natural gas, electricity); range 0–4.

d Heavy taste score: sum of dietary habits (high-oil food ≥1 time/week, pickled food ≥1 time/week, self-reported salt intake ≥ others); range 0–3.

### 3.2 Correlations between variables in SEM

Age demonstrated significant positive correlations with marital status, insurance status, ethnicity, male gender, smoking index, household air pollution exposure, and non-communicable disease (NCD) comorbidities (all *p* < 0.001), and negative correlations with educational attainment, household income, dietary salt preference (heavytaste), self-rated health, and health-related quality of life (HRQoL) utility scores (all *p* < 0.001). Marital status correlated positively with NCD comorbidities (*p* < 0.01) and negatively with educational attainment, household income, self-rated health, HRQoL utility scores (all *p* < 0.001), male gender (*p* < 0.05), and dietary salt preference (*p* < 0.01). Ethnicity showed positive correlations with household air pollution exposure (household fuel exposure), NCD comorbidity burden, HRQoL utility scores, and household income (all *p* < 0.001), and a negative correlation with alcohol use index (*p* < 0.001).

Educational attainment positively correlated with household income, dietary salt preference, and alcohol use index (all *p* < 0.001). Household income correlated positively with alcohol use index (*p* < 0.001). Dietary salt preference was positively associated with male gender (*p* < 0.05), alcohol use index, and smoking index (both *p* < 0.001). Male gender correlated positively with alcohol use index and smoking index (both *p* < 0.001). Alcohol use index correlated positively with smoking index (*p* < 0.001).

**Figure 2:**
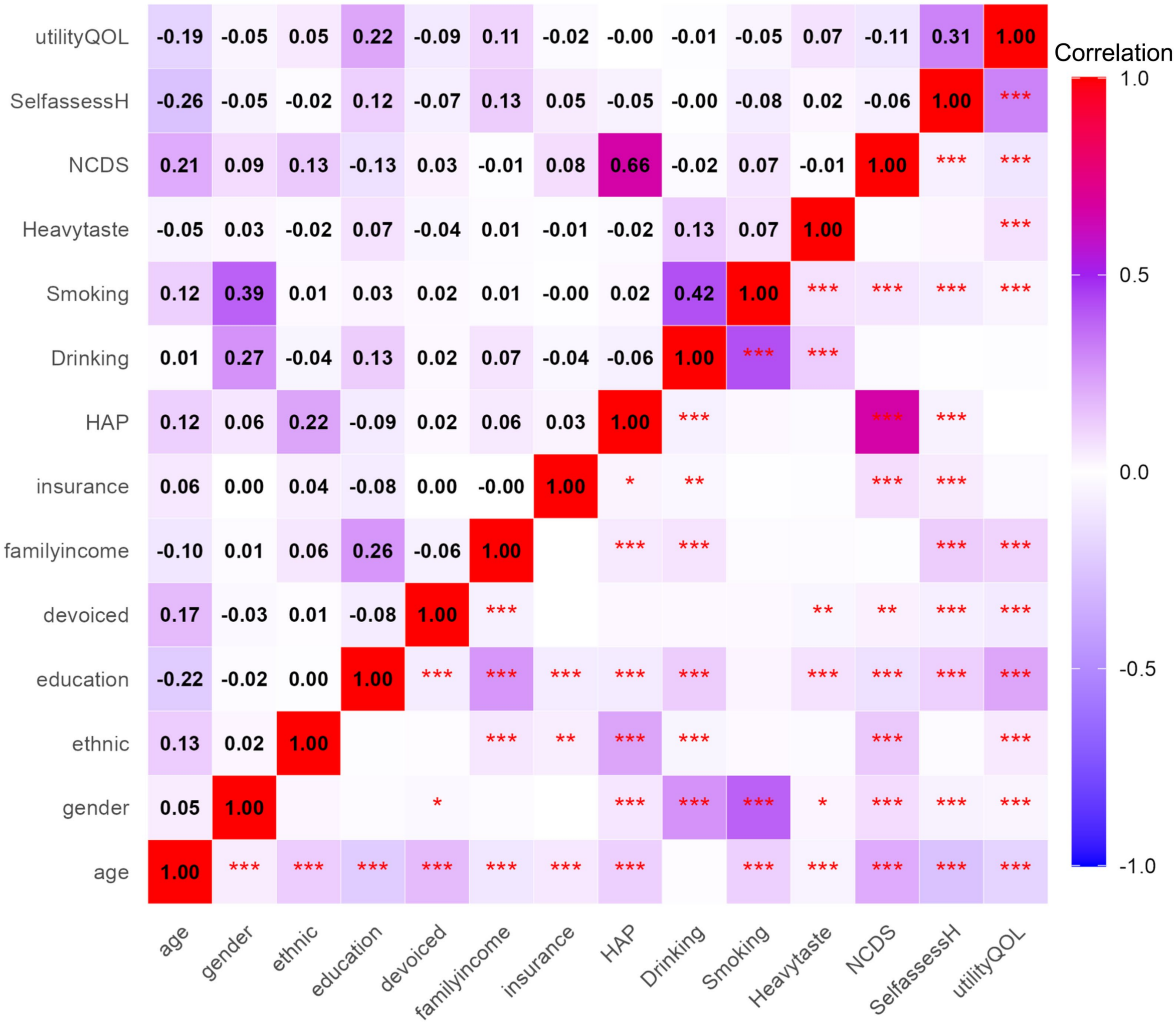
Correlations between variables in SEM model (UtilityQOL, utility values of quality of life; SelfassessH, self-assessed health ratings; NCDS, non-communicable disease comorbidity score; Heavytaste, heavy-taste score; Smoking, smoking index; Drinking, drinking index; HAP, household air pollution exposure; correlation matrix showing significant associations between variables; *p < 0.05, **p < 0.01, **p < 0.001).

Household air pollution exposure positively correlated with NCD comorbidities, household income, and male gender (all *p* < 0.001), and negatively with self-rated health, educational attainment, and alcohol use index (all *p* < 0.001). NCD comorbidity burden correlated positively with male gender and alcohol use index (both *p* < 0.001), and negatively with self-rated health, HRQoL utility scores, and educational attainment (all *p* < 0.001). Self-rated health positively correlated with HRQoL utility scores, household income, and educational attainment (all *p* < 0.001), and negatively with male gender and smoking index (both p < 0.001). HRQoL utility scores positively correlated with household income, educational attainment, and dietary salt preference (all p < 0.001), and negatively with male gender and smoking index (both p < 0.001) (Figure 2).

### 3.3 Factors associated with health related quality of life by SEM

**Figure 3:**
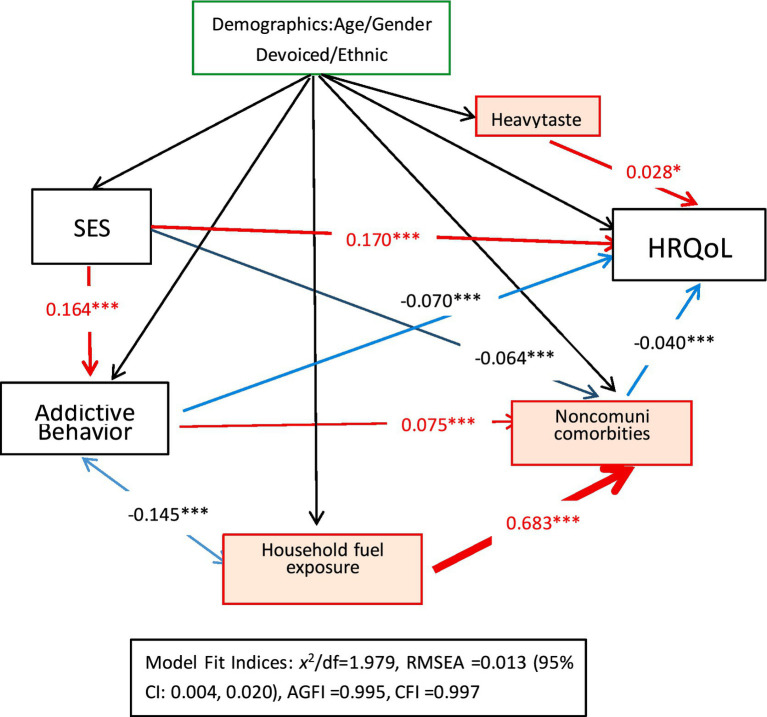
Pathways linking household air pollution, sociodemographic factors, dietary salt preference, NCD comorbidity burden, and health-related quality of life. For simplicity, the paths for demographic variables are not shown in detail but are included in the model. Path diagram with standardized coefficients; *p < 0.05, **p < 0.01, **p < 0.001.

The final structural model fitted the current data well [Chi-square = 31.664 (df = 16, *p* = 0.011), RMSEA = 0.013 (95% CI: 0.004, 0.020), AGFI = 0.995, and CFI = 0.997]. Figure 3 illustrates the associations between Health-Related Quality of Life (HRQoL), non-communicable diseases (NCDs), and associated factors.

#### 3.3.1 Direct effects

Several factors demonstrated significant direct associations with HRQoL. HRQoL was positively associated with socioeconomic status (SES) (direct effect *β* = 0.170, 95% CI: 0.140 to 0.199), ethnic majority status (direct effect *β* = 0.052, 95% CI: 0.027 to 0.077), and preference for high-salt/fat foods (direct effect *β* = 0.028, 95% CI: 0.006 to 0.051). Conversely, HRQoL was negatively associated with age (direct effect *β* = −0.171, 95% CI: −0.190 to −0.143), addictive behaviors (direct effect *β* = −0.070, 95% CI: −0.094 to −0.046), marital disruption (divorce/widowhood) (direct effect *β* = −0.082, 95% CI: −0.131 to −0.034), and NCDs (direct effect *β* = −0.040, 95% CI: −0.060 to −0.020).

Regarding factors associated with NCDs, the number of chronic conditions was positively associated with household air pollution exposure (direct effect *β* = 0.647, 95% CI: 0.615 to 0.679), age (direct effect *β* = 0.143, 95% CI: 0.123 to 0.162), and addictive behaviors (direct effect *β* = 0.075, 95% CI: 0.050 to 0.100). NCDs were negatively associated with higher SES (direct effect *β* = −0.064, 95% CI: −0.097 to −0.030) and ethnic majority status (direct effect *β* = −0.037, 95% CI: −0.064 to −0.010).

For the mediators, SES was positively associated with ethnic majority status (direct effect *β* = 0.242, 95% CI: 0.214 to 0.270) and negatively associated with age (direct effect *β* = −0.039, 95% CI: −0.067 to −0.010) and marital disruption (direct effect *β* = −0.117, 95% CI: −0.167 to −0.067). Addictive behaviors were positively associated with age (direct effect *β* = 0.043, 95% CI: 0.013 to 0.072) and male gender (direct effect *β* = 0.387, 95% CI: 0.360 to 0.414).

#### 3.3.2 Indirect effects via mediators

Beyond the direct associations, several factors exerted significant indirect effects on HRQoL through the mediators (NCDs, SES, and addictive behaviors). All indirect effects reported below are statistically significant based on bias-corrected bootstrap 95% confidence intervals.

Indirect effects mediated through NCDs: Age was indirectly associated with lower HRQoL via its positive association with NCDs (indirect effect *β* = −0.006, 95% CI: −0.009 to −0.003). Household air pollution exposure was indirectly associated with lower HRQoL via its positive association with NCDs (indirect effect *β* = −0.026, 95% CI: −0.039 to −0.013). Addictive behaviors were indirectly associated with lower HRQoL via their positive association with NCDs (indirect effect *β* = −0.003, 95% CI: −0.005 to −0.001). Higher SES was indirectly associated with higher HRQoL via its negative association with NCDs (indirect effect *β* = 0.003, 95% CI: 0.001 to 0.006).

Indirect effects mediated through SES: Age was indirectly associated with lower HRQoL via its negative association with SES, which in turn was positively associated with HRQoL (indirect effect *β* = −0.007, 95% CI: −0.012 to −0.001). Marital disruption (divorce/widowhood) was indirectly associated with lower HRQoL via its negative association with SES (indirect effect *β* = −0.020, 95% CI: −0.029 to −0.010).

Indirect effects mediated through addictive behaviors: Age was indirectly associated with lower HRQoL via its positive association with addictive behaviors (indirect effect *β* = −0.003, 95% CI: −0.005 to −0.001). Male gender was indirectly associated with lower HRQoL via its positive association with addictive behaviors (indirect effect *β* = −0.027, 95% CI: −0.005 to −0.001).

All reported effect sizes are standardized coefficients (β) with their corresponding 95% confidence intervals. Direct effects represent the unique association between two variables after accounting for all other paths in the model. Indirect effects represent the association between an exogenous variable and HRQoL that is transmitted through one or more mediating variables, calculated as the product of the constituent path coefficients.

## 4 Discussion

This study employed structural equation modeling (SEM) to elucidate the complex interplay between socioeconomic, behavioral, environmental, and clinical factors influencing Health-Related Quality of Life (HRQoL) in the context of non-communicable diseases (NCDs). The final model demonstrated excellent fit to the data, supporting the robustness of the proposed pathways.

Our findings underscore Socioeconomic Status (SES) as a factor strongly associated with HRQoL, exhibiting the strongest direct positive association (15). This aligns consistently with the global literature emphasizing SES as a fundamental driver of health disparities (26). Higher SES facilitates access to healthcare, healthier environments, nutritious food, and health literacy, all contributing to better HRQoL (27, 28). Crucially, SES was also associated with lower NCD burden (28) and mediated the detrimental effects of age and marital disruption on HRQoL (45). This highlights SES not only as a direct contributor but also as a critical buffer against other risk factors, reinforcing the concept of socioeconomic position as a root cause of health inequalities (15, 26).

Age was significantly associated with lower HRQoL and higher NCD burden (15, 16). The additional small but significant indirect effect of age on HRQoL via increased NCD risk illustrates the dual pathway through which aging is associated with well-being: directly through physiological decline and indirectly through the accumulation of chronic disease (16). This cumulative burden of aging and multimorbidity is a growing challenge for health systems globally (17).

Behavioral factors played a multifaceted role. Addictive behaviors (e.g., smoking, harmful alcohol use) were directly associated with HRQoL (46) and higher NCD risk (47). They also mediated negative effects on HRQoL from male gender and age (48). This positions addictive behaviors as a modifiable target for interventions aimed at improving both NCD prevention and HRQoL enhancement (48).

Our findings diverge from the established literature in two key aspects. First, contrary to prior evidence linking high-salt, high-fat, and pickled foods to increased chronic disease risk (39–42), we observed no significant association. This discrepancy may stem from differences in exposure assessment (self-reported preference vs. quantified intake), outcome definition (composite chronic diseases vs. site-specific conditions), or population characteristics. Second, the small positive association between dietary preference and HRQoL contradicts systematic reviews reporting detrimental effects of unhealthy dietary patterns on quality of life (43). Given the *E*-value of 1.13—indicating high susceptibility to unmeasured confounding—and the strong associations of socioeconomic status (SES) with both HRQoL (*β* = 0.170) and dietary preference (*β* = 0.066), this counterintuitive finding likely reflects residual confounding rather than a causal relationship. The observed effect may be partially attributable to inadequately controlled dimensions of SES (e.g., neighborhood environment or cumulative wealth), which could channel the strong SES-HRQoL association through dietary preference. Additionally, reverse causality (i.e., individuals with higher HRQoL may have greater opportunity to consume palatable foods) and cultural factors unique to our study population may contribute to these inconsistencies. Future studies employing longitudinal designs, objective dietary assessments, and comprehensive measurement of socioeconomic confounders are warranted to clarify these relationships.

Accumulating evidence links household air pollution (HAP) to chronic diseases, with the Global Burden of Disease Study 2021 estimating 111 million attributable DALYs worldwide, including type 2 diabetes (31). Specific associations have been documented for hypertension, diabetes, chronic kidney disease, and cardiovascular outcomes across diverse populations (29, 30, 33), while green-blue spaces appear protective via reduced pollutant exposure (32). Consistent with this evidence, HAP showed the strongest direct association with NCDs in our model (49), with substantial indirect negative effects on HRQoL mediated entirely through increased NCD burden (50). These findings underscore addressing indoor air quality as a public health priority, particularly in resource-limited settings (51), and support global efforts to transition to clean household energy (52).

Social demogriphic factors were also significant. Marital disruption (divorce/widowhood) was directly associated with HRQoL, likely reflecting the loss of social support and economic strain, an effect partially mediated by lower SES (18, 19). This aligns with global evidence that married individuals exhibit higher HRQoL than divorced/widowed groups across populations, including chronic disease patients and the older adults (18, 19). Ethnic majority status (Han in this context) showed a small positive association with HRQoL and a small inverse association with NCDs (20–23), contributing a minor positive indirect effect on HRQoL via reduced NCD risk (20, 23). This suggests potential advantages related to majority status (23), possibly through reduced discrimination or better access to culturally congruent services (53), although the effect sizes were modest (20, 21, 23).

NCD Burden itself was directly associated with lower HRQoL (54), serving as a key mediator translating upstream socioeconomic, behavioral, environmental, and demographic risks into impaired well-being (55). This mediating role highlights the critical importance of NCD prevention and management strategies as central to improving population HRQoL.

While the structural equation model revealed multiple statistically significant indirect pathways (e.g., age → NCDs → HRQoL: *β* = −0.006; addictive behaviors → NCDs → HRQoL: *β* = −0.003; marital disruption → SES → HRQoL: *β* = −0.020), the magnitude of these indirect effects warrants careful interpretation. With a large sample size (*N* = 5,978), even minimal effect sizes can achieve statistical significance; however, their practical or clinical significance may be limited. The indirect effects reported here range from −0.003 to −0.027, which are considered very small by conventional standards in behavioral and social sciences research. For instance, Gignac and Szodorai (56) proposed updated effect size guidelines for individual differences research, suggesting that correlations of 0.10, 0.20, and 0.30 represent small, typical, and large effects, respectively—benchmarks that are more conservative than Cohen’s original values and better reflect contemporary research distributions. By this standard, all indirect effects in our model fall well below the threshold for even a “small” effect. Funder and Ozer (57) further emphasized that the meaningfulness of effect sizes must be evaluated within the specific research context, cautioning against the mechanical application of universal thresholds. Moreover, with a large sample size (*N* = 5,978), even minimal effect sizes can achieve statistical significance—a phenomenon extensively discussed by Dick et al. (58) in the context of large-scale studies, where the critical question becomes whether effects are “practically important” rather than merely statistically detectable. The Primer on Effect Size Benchmarks (59) similarly provides field-specific benchmarks across multiple disciplines, reinforcing that the small effects observed here, while statistically significant, should be interpreted with caution regarding their population-level impact. These small effect sizes suggest that, although the hypothesized mediation pathways are statistically detectable, the amount of variance in HRQoL explained through these indirect routes is minimal. For example, an indirect effect of −0.006 indicates that the pathway (e.g., age increasing NCDs, which in turn lower HRQoL) accounts for less than 1% of the standardized difference in HRQoL. From a public health perspective, such small effects imply that intervening solely on these indirect pathways (e.g., targeting age-related NCD accumulation to improve HRQoL) would likely yield negligible population-level improvements unless the interventions are highly scalable and low-cost. Instead, the larger direct effects—particularly those of SES (*β* = 0.170) and age (*β* = −0.171) on HRQoL—represent more promising targets for intervention. Therefore, while the statistically significant mediation findings confirm the conceptual model and illustrate the interconnected nature of these determinants, their small magnitude tempers the enthusiasm for isolated, pathway-specific interventions. Future research should evaluate whether synergistic, multi-component interventions addressing multiple pathways simultaneously could produce clinically meaningful improvements in HRQoL that exceed the sum of these small individual effects (60).

### 4.1 Limitations and implications

While the model fit is excellent, cross-sectional data preclude causal inference. Self-reported measures (e.g., food preference, HAP) may be subject to bias. Moreover, mediation analysis in cross-sectional data can only suggest potential pathways and cannot establish temporal precedence or causal mediation; the estimated indirect effects should be interpreted as statistical associations rather than causal mechanisms. Nonetheless, this model emphasizes the necessity of integrated, multi-sectoral public health approaches that jointly address: (1) Socioeconomic inequalities as a fundamental cause; (2) Behavioral risk factors, particularly addictive behaviors; (3) Environmental health hazards, with priority given to reducing household air pollution (HAP) through clean energy transition strategies (e.g., subsidized access to electricity or biogas), improved stove technologies (e.g., efficient biomass stoves with chimneys), and structural ventilation improvements in dwellings; (4) NCD prevention and management; and (5) Social support systems, especially for older adults and those experiencing marital disruption. These multifaceted interventions, particularly those targeting HAP given its strong effect size in our study, are essential for mitigating disease burden in resource-limited settings.

## 5 Conclusion

Structural equation modeling identifies Socioeconomic Status (SES) as a key factor associated with better Health-Related Quality of Life (HRQoL) and lower NCD burden, directly associated with higher HRQoL and buffering age/marital disruption effects. Household air pollution (HAP) exposure showed the strongest negative association with HRQoL, largely through its link with increased NCD risk, while addictive behaviors were directly and indirectly (via NCDs) associated with lower HRQoL, mediating risks linked to male gender and age. Age was directly associated with lower HRQoL and increases NCD burden. Critically, NCD burden acts as the central mediator translating socioeconomic, behavioral, environmental, and demographic risks into diminished HRQoL. Findings highlight socioeconomic inequality as a fundamental correlate of disparities. Effective public health strategies require integrated, multi-sectoral interventions targeting SES determinants, HAP reduction, addictive behaviors, NCD prevention/management, and enhanced social support for vulnerable groups. Future longitudinal research is needed to confirm causality and evaluate interventions targeting these modifiable factors to improve population HRQoL and achieve health equity.

## Data Availability

The raw data supporting the conclusions of this article will be made available by the authors, without undue reservation.
